# Service quality and cross-cultural guest satisfaction in wellness tourism: evidence from yoga retreats in India’s global wellness destination

**DOI:** 10.12688/f1000research.177368.1

**Published:** 2026-04-16

**Authors:** Nikhil Singh Charak, Adorn Verma, Mahesh Uniyal, Jagadeesh Savanurmath, Nagendra Yadav, Neha Sharma, Ankit Shukla, Sanket Satish Shedbalkar

**Affiliations:** 1 Dr.B.R.Ambedkar University, Delhi, India; 2 GNA University, Phagwara, Punjab, India; 3 Assam Skill University, Mangaldoi, Assam, India; 4 Welcomgroup Graduate School of Hotel Administration, Manipal Academy of Higher Education, Manipal, India; 5 Welcomgroup Graduate School of Hotel Administration, Manipal Academy of Higher Education, Manipal, India; 6 Faculty of Hotel Management and Catering Technology, Parul University, Vadodara, Gujarat, India; 7 Faculty of Liberal Arts, Rajamangala University of Technology, Thanyaburi, Thailand; 8 Welcomgroup Graduate School of Hotel Administration, Manipal Academy of Higher Education, Manipal, India

**Keywords:** Wellness tourism; Service quality; Guest satisfaction; Cross-cultural differences; SERVQUAL; Expectation Confirmation Theory; Yoga retreats.

## Abstract

Wellness tourism is a rapidly growing global tourism segment, and India is recognised for its yoga, meditation, and holistic health traditions. Rishikesh, the “Yoga Capital of the World,” attracts wellness tourists seeking physical, mental, and spiritual well-being. Despite its importance, limited research has examined how perceptions of service quality and satisfaction differ between Indian and international guests at the same destination. This study investigates expectation perception gaps in service quality and their influence on satisfaction across culturally diverse groups. A quantitative, cross-sectional research design was adopted using an integrated SERVQUAL and Expectation–Confirmation Theory framework. Data were collected from 289 guests (178 Indians and 111 international travellers) at yoga retreats and spa resorts in Rishikesh using a structured questionnaire with a five-point Likert scale. Paired sample t-tests examined expectation perception gaps in service quality attributes, whereas independent t-tests assessed cross-cultural differences. Multiple regression and multi-group regression analyses identified satisfaction drivers and cultural effects. The results indicate that most service quality attributes met or exceeded expectations, showing a positive disconfirmation pattern across both Indian and international guests . However, significant cross-cultural differences were observed in the magnitude and importance of service quality dimensions. Indian guests reported greater satisfaction with empathy-based services, including staff courtesy and attention. In contrast, international guests emphasised tangible and professional attributes, such as spa quality, meditation effectiveness, environmental ambience, service efficiency, and cultural and spiritual experiences. Service quality dimensions explained a substantial variance in overall satisfaction, with greater explanatory power among international guests. The findings extend SERVQUAL and Expectation Confirmation Theory by demonstrating culturally moderated satisfaction formation in wellness tourism. This study provides insights for wellness destination managers, highlighting the importance of culturally differentiated service strategies to enhance satisfaction and sustain competitiveness in global wellness markets.

## 1. Introduction

Wellness tourism is a rapidly growing phenomenon worldwide, and India is making a bid to become one of the major destinations for wellness tourism due to its ancient, holistic health practices. Rishikesh is also known as the yoga capital of the world, where visitors come to practice yoga, meditate, and enjoy spa treatments. Although popular, empirical studies investigating the effects of service quality dimensions on the satisfaction of guests from different cultures are scarce ( Abdou et al., 2022). The four proposed objectives of this study are: (1) to measure the expectations-perception gap in quality of services between the Indian and international guests; (2) to determine the most important service quality attributes that affect overall customer satisfaction; (3) whether there are significant differences in service quality-satisfaction between domestic and international guests; and (4) to give recommendations to improve service quality and customer retention based on evidence ( Li et al., 2020).

The subsequent review synthesizes existing theories and empirical evidence on service quality and satisfaction in wellness and yoga tourism, incorporating SERVQUAL, the Expectation-Confirmation Theory, and frameworks on cultural variation to highlight knowledge gaps and justify the proposed conceptual model and hypotheses ( Leou & Wang., 2023; Dillette et al., 2019).

### 1.1 Satisfaction and service quality in wellness and yoga tourism

The significance of Satisfaction and Service Quality in Wellness and Yoga Tourism. Service quality has been recognized as a key determinant of guest satisfaction and behavioral intentions in hospitality and tourism studies. Service quality becomes even more critical in wellness and yoga tourism, as the experiences provided are often intangible, experiential, and transformative ( Ayvaz-Çavdaroğlu et al., 2024). The SERVQUAL model, developed by ( Parasuraman et al., 1988) is still one of the most used models of assessing the quality of service, which conceptualises the quality as the difference between customer expectations and perceptions in five dimensions, which include: tangibles, reliability, responsiveness, assurance, and empathy ( Berry et al., 1991).

Empirical studies within the context of wellness tourism continually indicate that expectation-perception gaps have a strong impact on overall satisfaction, loyalty, and revisit intentions. Research on spa resorts and wellness destinations suggests that physical factors, including facilities, cleanliness, and accommodation standards, interact with interpersonal dimensions, particularly empathy, assurance, and responsiveness, to influence guest service ratings ( Saleh & Ryan, 1991; Sangpikul, 2022a). Of all these dimensions, reliability and responsiveness have been most cited as the areas where negative disparities have occurred, leading to dissatisfaction when services are not delivered according to expected standards.

### 1.2 Core wellness services in the Indian wellness tourism context

Under the Indian wellness tourism scenario, yoga, Ayurveda, meditation, and spirituality are the main experiential elements for both domestic and international tourists. Such services are closely linked to the concepts of authenticity, professionalism, and holistic well-being, which, in turn, affect future behavioural intentions and levels of satisfaction ( Karagianni et al., 2025; Chhabra, 2021). The current body of literature confirms that an efficient provision of these services can improve satisfaction and increase revisit and recommendation intentions, demonstrating the strategic importance of service quality in the experiences of wellness tourism ( Alzoubi et al., 2021; Karagianni et al., 2025).

### 1.3 Service quality and behavioural intentions in wellness tourism

Service Quality and Behavioural Intent

Service quality, encompassing tangible and intangible factors and health and hygiene norms, has been identified as a pivotal driver of guest satisfaction and behavioural intentions in wellness and yoga tourism. Previous research indicates the importance of customised, individualised care and consideration of specific guest needs to improve satisfaction and create positive behavioural outcomes, such as revisit intentions and word-of-mouth recommendations ( Abdou et al., 2022; Kelly, 2012). The empirical data also show that service quality has a direct and indirect impact on satisfaction and loyalty, making it more relevant to the management of a wellness tourism setting ( Abdou et al., 2022).

### 1.4 Structural and competitiveness issues in Indian wellness tourism

The broader research on the ecosystem of wellness tourism in India focuses on structural and competitiveness-based issues ( Moirangthem & Nag, 2020). Applied Porter’s Diamond Model to determine the determinants of competitiveness in the Indian wellness industry, finding that infrastructure, service quality, and innovation were the primary drivers of competitiveness in the underdeveloped sector despite its high potential. On the same note, research on yoga tourism highlights the importance of better organisation, infrastructure, and marketing strategies to enhance India’s presence in international wellness markets ( Telej & Gamble, 2019).

### 1.5 Destination-specific evidence from Indian and Himalayan wellness resorts

Destination-specific studies also emphasise the importance of service quality in creating satisfaction and behavioural intentions. According to ( Charak, 2019) medicinal plants are a unique competitive advantage of spa and wellness tourism in India, and systematic development is necessary. Evidence-based studies on Himalayan wellness resorts show that discrepancies between guest expectations and service delivery, particularly regarding professionalism, ambience, and wellness services, significantly affect satisfaction and revisit intentions. The experiential nature of satisfaction is also supported by qualitative studies showing that yoga tourists are primarily driven by physical and spiritual health, stress reduction, and a desire to enhance their lives in their choices ( Karagianni et al., 2025).

### 1.6 Interpersonal relationships and experiential dimensions of wellness services

Interpersonal relationships are critical in wellness experiences, and the interaction between therapists and clients has become an important factor in spa and wellness experiences ( Kajornatthapol et al., 2024). Cross-theoretical strategies also indicate that the effects of perceived service quality, cultural and spiritual experiences, and confirmed expectations on satisfaction and revisit intentions depend on interactions among these aspects among yoga tourists ( Leou & Wang, 2023). Taken together, the literature confirms that enhancing service quality and eliminating structural gaps are needed to maintain behavioural loyalty in the wellness tourism industry in India ( Datta, 2022).

## 2. Theoretical framework

### 2.1 Expectation–confirmation theory and satisfaction formation

The original Theory of Expectation-Confirmation Theory and Satisfaction Formation Expectation-Confirmation Theory (ECT), which was first suggested by ( Oliver, 1980) offers a strong theoretical basis to the formation of satisfaction in the tourism setting ( Majeed et al., 2020). ECT proposes that satisfaction results from comparing post-consumption performance perceptions with pre-consumption expectations. Positive confirmation occurs when an individual feels satisfaction because the performance is perceived as meeting or exceeding expectations, whilst negative confirmation causes dissatisfaction.

The rationale of ECT conforms well to the SERVQUAL framework that operationalises satisfaction in terms of expectation-perception gaps. It has become a dominant feature of tourism studies with special emphasis on experiential tourism, including wellness and yoga tourism ( Sangpikul, 2022b). By the time they arrive at a wellness retreat, these visitors to wellness resorts have high expectations regarding personal transformation, stress relief, and spiritual development, and so even slight failures in service provision could result in negative disconfirmation ( Chhabra & Kim, 2024). As a result, to maintain satisfaction in these environments, service stability is necessary.

The empirical findings demonstrate the applicability of expectation-perception discrepancies in predicting levels of satisfaction and intentions to engage in wellness tourism, and they justify the use of ECT in elucidating the process by which service experiences are converted into post-consumption judgments. It is a theoretical angle that is quite applicable to comparative studies of the satisfaction patterns of various visitor groups ( Uysal et al., 2015).

## 3. Cross-cultural perspectives on service quality and satisfaction

Cross-Cultural Approach to Service Quality and Satisfaction. Cross-cultural theories underscore that cultural background plays an influential role in expectations, perceptions, and ratings of the influence of service quality on tourists. Differences in values, norms, and consumption behaviours will affect how guests perceive service encounters and determine their satisfaction. In hospitality research, it is consistently demonstrated that international and domestic tourists do not have the same priorities in their service feature preferences: international visitors often place greater emphasis on authenticity, customisation, and cultural immersion, whereas domestic visitors are more interested in functional efficiency and familiarity ( Li et al., 2024).

The value-satisfaction-loyalty model also implies that perceived value and satisfaction are crucial antecedents of loyalty-related behavioural tendencies, such as revisit intentions and positive word of mouth ( Armstrong et al., 1997). This model can be applied to wellness tourism by showing how perceptions of service quality can be translated into long-term behavioural outcomes through satisfaction mechanisms ( Azimi et al., 2025).

Although there is an increasing body of literature on wellness and yoga tourism in India, most extant work either focuses on international tourists or treats them as a homogeneous group. Direct comparative studies examining the effects of service quality dimensions on the satisfaction of domestic and international guests are still scarce. This disconnect is particularly notable in destination-specific contexts, such as Rishikesh, where different visitor segments can be found within the same service environment ( Kumar, 2017).

## 4. Research gap and hypotheses development

The literature review identifies three major gaps. To begin with, there is a paucity of empirical research comparing the satisfaction drivers and service quality perceptions of Indian and international visitors in the same wellness destination. Second, only a merger effort has been made to determine the service quality dimensions with the strongest predictive power for each visitor group. Third, there is a paucity of context-specific evidence on globally recognised yoga destinations such as Rishikesh ( Leou & Wang, 2023).

To fill these gaps, the current research paper will use an integrated SERVQUAL-ECT model to examine the absence of the expectation-perception gap, satisfaction determinants, and cross-cultural differences between Indian and international tourists visiting yoga retreats and spa resorts in Rishikesh ( Mishra & Panda, 2021).

### 4.1 Empirical basis for hypotheses formulation

Empirical findings in Indian wellness tourism indicate that tangible components with a significant impact on perceived service quality include facilities, food quality, accommodation, and hygiene, which are among the most frequent points of dissatisfaction when expectations are unmet ( Manhas & Tukamushaba, 2015). There are also significant discrepancies in the interpersonal aspects of empathy, assurance, reliability, and responsiveness, especially when employees are perceived as unattentive or inefficient ( Mishra & Panda, 2021). Sentiment analysis also reveals that negative experiences are often linked with a lack of assurance, empathy, and reliability.

### 4.2 Hypotheses development

Research on cross-cultural hospitality proves that the level of expectation-perception discrepancies depends on the cultural backgrounds, previous experiences, and evaluative criteria of the visitors ( Armstrong et al., 1997; Ayyıldız, 2024; Carreón et al., 2021). According to evidence from Asian and international tourism contexts, it can be assumed that such discrepancies are stronger in culturally heterogeneous settings ( Carreón et al., 2021). Direct comparative studies are scarce in Rishikesh; still, based on other Indian and Himalayan wellness centres, there is a likelihood that cultural diversity will lead to differences in service quality perceptions between domestic and international guests ( Mishra & Panda, 2021).

H1: There are significant differences between the expectations and perceptions of the guests in the dimensions of the service quality of yoga retreats and spa resorts in Rishikesh between international and Indian tourists.

Other earlier studies also find that the dimensions of service quality have a cumulative effect on overall guest satisfaction in wellness tourism. Among the key predictors of satisfaction in spa resorts and yoga retreats, tangibles, reliability, responsiveness, assurance, empathy, health, and hygiene have been identified ( Mishra & Panda, 2021; Ali et al., 2021; Abdou et al., 2022). These dimensions also contribute to positive behavioural intentions, such as revisit intentions and favourable word of mouth ( Abdou et al., 2022; González et al., 2007).

H2: Service quality dimensions have a significant overall influence on guest satisfaction in both yoga retreats and spa resorts in Rishikesh.

Lastly, there is no uniform impact of service quality dimensions on satisfaction across different visitor groups. Cultural background and nationality mediate satisfaction by influencing guests’ perceptions of service attributes ( Matzler et al., 2006; Al-Ansi et al., 2025). Empirical research indicates that international visitors are more likely to be sensitive to tangibles and reliability, whereas domestic visitors are more likely to be sensitive to empathy and comfort ( Matzler et al., 2006). Nationality, hence, enters the service quality-satisfaction connection in wellness tourism ( Vryoni et al., 2017).

H3: The relations between the dimensions of service quality and overall guest satisfaction are significantly different in terms of Indian and international guests.

Table 1 synthesises the main themes of the literature on wellness tourism and identifies gaps in the research that form the basis of the current project. The extant literature extensively covers international wellness tourists and identifies physical, spiritual, and affordability factors that drive their motivations, but the Indian market has been relatively under-researched. Furthermore, the discussion of the direct association between guests’ motivations and satisfaction in the domestic and international segments is rarely addressed in previous studies.

**Table 1.** Summary of comparative insights and research gaps for Indian vs. International Guests.

Key Focus	Literature known	Gap	Citations
Guests Motivations	International research related to physical, spiritual, and affordability where whereas the Indian context is less studied	Direct relationship of motivations	( Singh & Ansari, et al., 2024; Datta, 2022; Charak et al., 2021)
Satisfaction elements	Core wellness services, authenticity, and emotional experience	Attribute-level analysis by visitor type	( Datta, 2022; Sharma & Nayak, 2019; Abdou et al., 2022)
Quality of Service	Impacts satisfaction and intentions	Comparative analysis of perceptions	( Abdou et al., 2022)
Rishikesh	Recognized as a yoga hub	Tailored, comparative recommendations	( Leou & Wang, 2023)

Considering the aspects of satisfaction, core wellness services, authenticity and emotional experiences have been cited by the literature as the key determinants of satisfaction. Nevertheless, there is no attribute-level and visitor-type-specific analysis that hinders the comprehension of the impact of various elements of service on satisfaction in different cultures. Likewise, although it is clearly known that service quality is a determinant of satisfaction and intent to act, there is a dearth of comparative studies on the differences in perception of Indian and international customers in the same destination.

Even though the scope of the current research is rather limited, and despite Rishikesh being established as a world-renowned centre of yoga and wellness, there are few supportable, culturally-focused managerial suggestions available. All in all, Table 1 confirms that comparing attribute-specific and cross-cultural research on the quality of service and customer satisfaction is necessary, hence the purpose of the current research and its design.

### 4.3 Comparative insights and gaps

### 4.4 Research methodology

The research design used was a quantitative, cross-sectional, comparative study aimed at identifying gaps in service quality, predictors of satisfaction, and the cross-cultural impacts of wellness tourism. Purposive stratified sampling was used to select five yoga retreats and spa resorts in Rishikesh, India (N = 289; 178 Indian, 111 international guests) and mostly middle-aged professionals (mean age 36–45, 61.6 per cent female, 58.5 per cent first-time visitors). A scaled SERVQUAL was used to determine the 5 dimensions: Tangibles, Reliability, Responsiveness, Assurance, and Empathy that required a comparison between expectations and perceptions on a 5-point Likert scale. Paired and independent t-tests, one-way ANOVA, and standardised coefficients were analysed. Before participating, all respondents were fully informed about the study’s aims and objectives. This study did not involve any sensitive personal data collection, clinical procedures, or interventions. This research was designed and conducted in accordance with the ethical principles of the Declaration of Helsinki. According to institutional norms and guidelines for social science research, ethical approval was not required for this type of study. No personally identifiable information was collected at any stage, ensuring respondents’ complete anonymity. Prior to data collection, informed consent was obtained from all participants. A written consent checkbox was provided at the start of the online questionnaire, clearly stating the study’s purpose, the voluntary nature of participation, the confidentiality of responses, and the exclusive use of the data for academic research. Participants were explicitly informed that they could discontinue participation at any time without justification, and that no data would be retained if they chose to withdraw. These measures ensured adherence to ethical standards for research involving human respondents.

### 4.5 Data analysis

Table 2 shows the demographic and travel attributes of 289 participants who were used in the study, and it gives a view of the sample demographics employed in the cross-cultural study of service quality perception and satisfaction. The sample size consists mostly of Indian guests (61.6%), with the international guests making up 38.4, which is a very wide-ranging sample representing several countries such as the United States, United Kingdom, Germany, Australia, Canada and various Asian and European countries. The distribution guarantees that there is an appropriate representation of both domestic and foreign wellness tourists and that there is a sound comparative analysis between the two.

**Table 2.** Demographic.

Demographic variable	n	Percentage (%)
**Total Sample**	289	100
** Indian Guests**	178	61.6
**International Guests (**United States, United Kingdom, Germany, Australia, Canada, France, Netherlands, United Arab Emirates (UAE), New Zealand, Italy, Spain, Switzerland, Belgium, Israel, Japan, South Korea, Singapore, Thailand)	111	38.4
**Age Distribution**		
18–25 years	33	11.4
26–35 years	87	30.1
36–45 years	90	31.1
46–55 years	79	27.3
56+ years	-	-
**Gender**		
Female	178	61.6
Male	111	38.4
**Occupation**		
Business	115	39.8
Government Employees	86	29.8
Private Sector	61	21.1
Retired	27	9.3
**Monthly Income (INR)Lakhs**		
Below 1 Lakh	23	8
1–2 Lakhs	66	22.8
2–5 Lakhs	133	46
Above 5 Lakhs	67	23.2
**Visit History**		
First-time Visitors	169	58.5
Repeat Visitors	120	41.5

Regarding age, most of the respondents are aged between 26 and 45 years, and that is more than 61% of the sample. In particular, 31.1% of them are between 36–45 years and 30.1% between 26–35 years, with the next 27.3-year-old age group. The younger visitors between the ages of 18 and 25 years are the only ones, 11.4, and the rest of the respondents were aged over 56 years. This trend indicates that middle-aged adults who are generally health-conscious, financially secure and leaner towards restorative or preventive wellness experiences are the main participants of wellness tourism in Rishikesh.

The gender distribution shows that the female guests are more active (61.6%) than males (38.4%), which means that the female guests are likely to be more active in yoga, meditation, and holistic wellness practices. It is consistent with the tendencies in wellness and retreat tourism, where female customers tend to show more interest in self-care, stress relief, and experiential healthcare services.

The sample occupationally comprises predominantly business people (39.8%) and government workers (29.8%), then there are the employees of the private sector (21.1) and retirees (9.3). This profile displays an industrious and economically active citizenship, having organised lifestyles, and probably would desire wellness retreats as a way of relieving stress and balancing work and life. This interpretation is further confirmed by the levels of income distribution, with almost 69 percent of the respondents having an average of 2–5 or above 5 lakhs income per month shown by the distribution levels of income. This kind of financial ability implies that the respondents can afford high-quality wellness services, and this aspect might affect their expectations of the quality and professionalism of the services.

In terms of visit history, most of the respondents (58.5%) are first-time visitors, with a good share (41.5%) being return customers. The high number of repeat visitors shows that there is a positive experience with the overall destination and that there is a possibility of being loyal and satisfied with the services provided to them. Meanwhile, the high proportion of first-time consumers indicates that Rishikesh remains popular among new wellness tourists.

The demographic profile, in general, shows that the study sample is quite heterogeneous but consists mostly of middle-aged, financially endowed women and professionally employed people, which is quite appropriate in the context of testing service quality expectations and satisfaction drivers in wellness tourism. Such characteristics give a good basis to further comparative and inferential studies.

Having reached a descriptive analysis, the inferential analysis was applied to investigate differences between perceptions and actual impressions of the services provided to guests in Rishikesh. To accomplish this, 15 statements concerning services offered by these yoga retreats and spas were subjected to a paired t-test.

Table 3 illustrates the Paired samples t-tests examining expectation-perception gaps across 15 service attributes revealed that 12 of 15 attributes (86.7%) demonstrated statistically significant differences between guest expectations and actual experiences (p < 0.01), with all attributes showing positive mean gaps ranging from 0.02 to 0.78, indicating guests’ experiences met or exceeded expectations across both Indian (n = 178) and international (n = 111) guest groups. Only yoga instruction quality (t = 0.419, p = 0.676; Indian M = 0.03, SD = 1.12; International M = 0.02, SD = 1.1) and meal quality (t = 1.718, p = 0.087; Indian M = 0.14, SD = 1.57; International M = 0.17, SD = 1.58), yoga styles and programmes (p = 0.428) showed non-significant gaps, confirming these core wellness services reliably met both cultural groups’ expectations and representing universal satisfaction drivers transcending cultural boundaries. Cross-cultural moderation analysis revealed significant differences in expectation-perception gaps between Indian and international guests on 12 attributes, supporting hypotheses of cultural moderation effects. Staff courtesy and attentiveness showed substantially larger gaps for Indian guests (M = 0.61, SD = 1.23) than for international guests (M = 0.55, SD = 1.28; t = 7.868, p < 0.01), confirming that the Empathy dimension is more important for Indian guest satisfaction, reflecting the collectivist cultural emphasis on interpersonal warmth and personal relationships. Conversely, international guests reported significantly larger positive disconfirmation on scenic setting (t = 10.384, p < 0.01; International M = 0.78 vs. Indian M = 0.75), meditation program effectiveness (t = 7.758, p < 0.01; International M = 0.63 vs. Indian M = 0.58), customized wellness programs (t = 3.374, p < 0.01; International M = 0.30 vs. Indian M = 0.27), reservation/check-in efficiency (t = 5.315, p < 0.01; International M = 0.38 vs. Indian M = 0.32), recreational facilities (t = 4.096, p < 0.01; International M = 0.31 vs. Indian M = 0.25), cultural-spiritual experiences (t = 5.603, p < 0.01; International M = 0.38 vs. Indian M = 0.36), and post-retreat wellness guidance (t = 7.009, p < 0.01; International M = 0.45 vs. Indian M = 0.42), predicting that Reliability, Responsiveness, and observable service attributes are more important for international guest satisfaction. Cleanliness standards (t = 2.612, p < 0.01; International M = 0.21 vs. Indian M = 0.16) and room comfort (t = 3.398, p < 0.01; International M = 0.33 vs. Indian M = 0.29) showed modest but significant international guest advantages, reflecting international guests’ higher expectations for visible quality standards and tangible amenities. Value perception was positive for both groups (Indian M = 0.40, SD = 1.09; International M = 0.43, SD = 1.1; t = 6.417, p < 0.01), indicating pricing strategies are well-calibrated and perceived as justified by both cultural groups. Collectively, these results demonstrate that while all service dimensions met or exceeded guest expectations (positive disconfirmation pattern), the magnitude of expectation-perception gaps differs significantly between cultural groups, with Indian guests experiencing greater positive disconfirmation regarding interpersonal service dimensions (staff warmth, personalization) and international guests experiencing greater positive disconfirmation regarding tangible, observable dimensions (scenery, facilities, efficiency, cultural experiences), validating cross-cultural moderation of service quality-satisfaction relationships and supporting the theoretical framework integrating SERVQUAL dimensions with Expectation-Confirmation Theory and Hofstede’s cultural dimensions theory ( Jingyun et al., 2007). These differentiated satisfaction drivers have direct implications for market-segmented service strategies, suggesting management should prioritize staff empathy training and personalized service for Indian guest markets while prioritizing environmental enhancement, digital efficiency, cultural immersion programming, and post-retreat continuity for international guest markets, while maintaining universal standards for core services (yoga instruction, meals, cleanliness) to ensure baseline satisfaction across all guest segments ( Emara et al., 2023).

**Table 3.** Paired t-test statistics for differences between guests ‘perceptions and actual experiences of the services offered at yoga retreats and spas.

Service attribute	Indian guests (n = 178)		International guests (n = 111)		t-value	Sig. (2-tailed)	Significance	Interpretation
	Mean	SD	Mean	SD				
The yoga sessions are well-structured and led by experienced instructors	0.03	1.12	0.02	1.1	0.419	0.676	Not significant	No significant difference: both groups were equally satisfied with yoga instruction.
The resort offers a diverse range of yoga styles and programs	0.06	1.11	0.05	1.08	0.793	0.428	Not significant	Both groups perceived the variety of yoga similarly; expectations were well met.
The spa treatments are relaxing, professional, and meet expectations	0.57	1.05	0.72	1.1	9.891	0	**p** < 0.01	International guests rated the spa quality higher; their expectations were more likely to be met.
The resort maintains high standards of cleanliness in rooms, yoga halls, and common areas	0.16	1.18	0.21	1.25	2.612	0.009	**p** < 0.01	International guests reported slightly higher gaps; stricter cleanliness benchmarks.
The rooms are comfortable, well-equipped, and provide a peaceful ambiance	0.29	1.53	0.33	1.55	3.398	0.001	**p** < 0.01	Both groups rated rooms positively; international guests had slightly higher satisfaction.
The meals provided are healthy, fresh, and cater to dietary preferences	0.14	1.57	0.17	1.58	1.718	0.087	Not significant	No significant difference: meal quality met the expectations of both groups.
The staff members are courteous, helpful, and attentive to guests needs	0.61	1.23	0.55	1.28	7.868	0	**p** < 0.01	Domestic guests rated staff more positively; interpersonal warmth was valued.
The resort is in a peaceful and scenic setting, enhancing relaxation	0.75	1.26	0.78	1.25	10.384	0	**p** < 0.01	International guests placed higher importance on scenic serenity.
The meditation and mindfulness programs are effective and enriching	0.58	1.3	0.63	1.35	7.758	0	**p** < 0.01	International guests valued mindfulness depth more strongly.
The resort provides customized wellness programs based on individual needs	0.27	1.41	0.3	1.45	3.374	0.001	**p** < 0.01	International guests expected higher personalization, a minor but significant difference.
Additional recreational facilities (e.g., swimming pool, nature walks) are well-maintained	0.25	1.18	0.31	1.21	4.096	0	**p** < 0.01	International guests had higher leisure expectations; the perception gap was larger.
The reservation and check-in process is smooth and hassle-free	0.32	1.13	0.38	1.15	5.315	0	**p** < 0.01	International guests reported greater digital service expectations.
The services provided justify the overall cost of the stay	0.4	1.09	0.43	1.1	6.417	0	**p** < 0.01	Both groups were satisfied with the value; international guests were slightly more critical.
The resort offers enriching cultural and spiritual experiences (e.g., Ganga Aarti, temple visits)	0.36	1.12	0.38	1.13	5.603	0	**p** < 0.01	International guests valued cultural immersion more highly.
The resort provides guidance and resources for continuing yoga and wellness practices after departure	0.42	1.06	0.45	1.07	7.009	0	**p** < 0.01	International guests appreciated post-retreat wellness continuity slightly more.

Furthermore, to examine the relationship between the services provided at the Yoga Retreat and Spa Resort in Rishikesh and guests’ overall satisfaction, a multiple regression analysis was conducted. The analysis included 15 independent variables representing different aspects of the resort’s services and one dependent variable: guests’ level of satisfaction.

The model summary indicated that the regression model had a strong predictive capability, with an R value of 0.840, suggesting a high correlation between the independent variables and guest satisfaction. The R-square value was 0.705, indicating that 70.5% of the variance in guest satisfaction could be explained by the services provided. The adjusted R-square was 0.689, which is consistent with the number of predictors in the model, confirming strong explanatory power. The standard error of the estimate was 0.31173, and the Durbin-Watson statistic was 1.772, indicating no significant autocorrelation in the residuals ( Colonescu, 2016).

The residual statistics confirmed the reliability of the regression model. The mean of predicted values was 4.1869, with a standard deviation of 0.46912. The residuals ranged from -0.66233 to 0.68993, with a mean of 0.00000 and a standard deviation of 0.30351, indicating a symmetric distribution around zero. The standardised predicted values ranged from -4.080 to 1.837, and the standardised residuals ranged from -2.125 to 2.213, suggesting no major outliers affecting the model’s accuracy.

The ANOVA results further confirmed the model's significance. The regression sum of squares was 63.380, with 15 degrees of freedom, while the residual sum of squares was 26.530 with 273 degrees of freedom, leading to a total sum of squares of 89.910. The F-value was 43.480, and the significance value (Sig.) was 0.000, indicating that the overall regression model was statistically significant.

The coefficients in Table 4 provided further insight into the contribution of each independent variable to the model. The constant term was -0.646, with a standard error of 0.207, and was statistically significant (t = -3.121, p = 0.002). Several independent variables had significant positive effects on guest satisfaction. Among them, the most influential predictor was “The spa treatments are relaxing, professional, and meet expectations” (B = 0.126, t = 4.120, p = 0.000), followed by “The yoga sessions are well-structured and led by experienced instructors” (B = 0.123, t = 5.020, p = 0.000). Other significant predictors included “The resort provides guidance and resources for continuing yoga and wellness practices after departure” (B = 0.117, t = 4.196, p = 0.000), “The meditation and mindfulness programs are effective and enriching” (B = 0.105, t = 4.098, p = 0.000), and “The reservation and check-in process is smooth and hassle-free” (B = 0.105, t = 3.800, p = 0.000). These results indicate that structured yoga sessions, wellness guidance, mindfulness programs, and professional spa treatments were key factors in enhancing guest satisfaction.

**Table 4.** Unstandardized and standardized beta coefficients for predictors of guest satisfaction.

Model		B	Std. error	Standardised Beta	t	Sig.
**1**	(Constant)	−0.646	0.207		−3.121	0.002
	The yoga sessions are well-structured and led by experienced instructors	0.123	0.024	0.182	5.020	0.000
	The resort offers a diverse range of yoga styles and programs	0.087	0.026	0.132	3.396	0.001
	The spa treatments are relaxing, professional, and meet expectations	0.126	0.030	0.150	4.120	0.000
	The resort maintains high standards of cleanliness in rooms, yoga halls, and common areas	0.050	0.022	0.082	2.229	0.027
	The rooms are comfortable, well-equipped, and provide a peaceful ambiance	0.059	0.017	0.145	3.426	0.001
	The meals provided are healthy, fresh, and cater to dietary preferences	0.071	0.018	0.170	3.867	0.000
	The staff members are courteous, helpful, and attentive to guests needs	0.085	0.024	0.133	3.561	0.000
	The resort is in a peaceful and scenic setting, enhancing relaxation	0.049	0.027	0.072	1.782	0.076*
	The meditation and mindfulness programs are effective and enriching	0.105	0.026	0.166	4.098	0.000
	The resort provides customized wellness programs based on individual needs	0.034	0.018	0.077	1.897	0.059*
	Additional recreational facilities (e.g., swimming pool, nature walks) are well-maintained	−0.010	0.023	−0.018	−0.447	0.656*
	The reservation and check-in process is smooth and hassle-free	0.105	0.028	0.148	3.800	0.000
	The services provided justify the overall cost of the stay	0.085	0.029	0.110	2.927	0.004
	The resort offers enriching cultural and spiritual experiences (e.g., Ganga Aarti, temple visits)	0.055	0.028	0.079	1.973	0.049
	The resort provides guidance and resources for continuing yoga and wellness practices after departure	0.117	0.028	0.152	4.196	0.000

Additionally, “The rooms are comfortable, well-equipped, and provide a peaceful ambiance” (B = 0.059, t = 3.426, p = 0.001), “The staff members are courteous, helpful, and attentive to guest needs” (B = 0.085, t = 3.561, p = 0.000), and “The meals provided are healthy, fresh, and cater to dietary preferences” (B = 0.071, t = 3.867, p = 0.000) were also significant predictors, suggesting that cleanliness, hospitality, and food quality played a crucial role in shaping overall guest satisfaction.

However, some predictors were not statistically significant. “The resort provides customized wellness programs based on individual needs” (B = 0.034, t = 1.897, p = 0.059) and “The resort is located in a peaceful and scenic setting, enhancing relaxation” (B = 0.049, t = 1.782, p = 0.076) had p-values slightly above the 0.05 threshold, indicating that while they contributed to satisfaction, their impact was not as strong as other factors. Additionally, “Additional recreational facilities (e.g., swimming pool, nature walks) are well-maintained” had a negative coefficient (B = -0.010, t = -0.447, p = 0.656), suggesting that it did not significantly influence guest satisfaction.

In conclusion, the multiple regression analysis revealed that several factors significantly influenced guest satisfaction at the Yoga Retreat and Spa Resort in Rishikesh. The most impactful predictors included structured yoga sessions, professional spa treatments, effective mindfulness programs, high-quality meals, and courteous staff service. Other aspects, such as the peaceful ambience of rooms, cleanliness, and smooth check-in processes, also contributed positively. The findings suggested that enhancing the key services identified in the analysis can further improve the guest experience at the resort. These findings also validate and extend both the SERVQUAL framework (confirming service quality dimensions predict satisfaction) and Expectation-Confirmation Theory (confirming importance-weighted satisfaction formation), while empirically demonstrating that management efficiency requires not merely ensuring general service quality across all dimensions, but rather strategic prioritisation of investments in core wellness services where coefficient magnitudes demonstrate substantially higher satisfaction-generation potential, thereby offering concrete, evidence-based guidance for management decision-making regarding service quality resource allocation in competitive wellness tourism markets ( Karagianni et al., 2025; Phuthong et al., 2022).

The hypothesis that relationships between service quality dimensions and overall guest satisfaction differ significantly between Indian and international guests is substantially supported in Table 5, with multi-group regression analysis demonstrating meaningful cross-cultural moderation effects wherein the standardised coefficient magnitudes and patterns systematically diverge between cultural groups across 13 of 15 service attributes, indicating that cultural background significantly moderates which service dimensions drive satisfaction. The regression model revealed markedly different explanatory power between cultural groups. The model explained substantially more satisfaction variance for international guests (R ^2^ = 0.76) than for Indian guests (R ^2^ = 0.71), a 5-percentage-point difference suggesting that service quality-satisfaction relationships are more tightly coupled and more systematically organised for international guests, potentially reflecting their greater reliance on service quality. Core wellness services demonstrated the strongest cross-cultural differentiation, with yoga instruction quality showing stronger effects for international guests (β_International = 0.198 vs. β_Indian = 0.161, β-difference = 0.037), meditation programs similarly showing stronger international relationships (β_International = 0.172 vs. β_Indian = 0.153, β-difference = 0.019), and spa treatments demonstrating marked international guest advantage (β_International = 0.172 vs. β_Indian = 0.136, β-difference = 0.036), collectively suggesting that international guests weight core wellness service quality more heavily in satisfaction judgments, possibly reflecting their higher expectations for professional service standards and instruction quality. Conversely, interpersonal service dimensions demonstrated the opposite pattern, with staff courtesy showing substantially stronger effects for Indian guests (β_Indian = 0.141 vs. β_International = 0.105, β-difference = −0.036), providing strong empirical evidence that the Empathy dimension is more important for Indian guests who prioritise relationship-building and personal warmth in service interactions. Supporting infrastructure services showed relatively equivalent effects across groups (rooms: β_Indian = 0.144 vs. β_International = 0.139, β-difference = −0.005; meals: β_Indian = 0.161 vs. β_International = 0.168, β-difference = 0.007; check-in: β_Indian = 0.155 vs. β_International = 0.151, β-difference = −0.004), indicating that these foundational services are universally important satisfaction drivers, unmoderated by cultural background. Peripheral tangible attributes revealed consistent international guest advantages across all dimensions: scenic setting (β-difference = 0.031), cultural experiences (β-difference = 0.022), and cleanliness (β-difference = 0.007), suggesting international guests place higher importance on observable environmental quality and experiential variety, consistent with individualistic cultural values emphasising explicit, visible quality indicators. The cross-cultural coefficient patterns validate Hofstede’s cultural dimensions theory, in which collectivist Indian guests prioritise relational service dimensions (staff warmth, personal attention) while individualistic international guests prioritise professional, efficient, observable service quality (yoga expertise, spa professionalism, environmental cleanliness, cultural programming). Collectively, these multi-group regression findings provide decisive empirical support for Hypothesis H3, demonstrating that satisfaction formation mechanisms differ substantially between cultural groups such that management strategies cannot employ uniform service quality approaches across markets successful satisfaction management for Indian guests requires emphasis on staff empathy training, personalized service, and relationship-building, while successful satisfaction management for international guests requires emphasis on professional expertise, observable quality standards, environmental enhancement, and cultural mmersion experiences, thereby offering strategic guidance for market-differentiated service quality management in culturally diverse wellness tourism contexts ( Pan et al., 2024; Phuthong et al., 2023).

**Table 5.** Unstandardized and standardized beta coefficients for predictors of guest satisfaction amongst Indian and international guests.

Service attribute	Indian guests (n = 178)			International guests (n = 111)			β (Difference)	Interpretation
	B	β	Sig.	B	β	Sig.		
The yoga sessions are well-structured and led by experienced instructors	0.102	0.161	0.002	0.145	0.198	0	0.037	Stronger predictor for international guests.
The resort offers a diverse range of yoga styles and programs	0.071	0.121	0.018	0.095	0.136	0.003	0.015	Slightly higher impact among international guests.
Spa treatments are relaxing, professional, and meet expectations	0.113	0.136	0.006	0.144	0.172	0.001	0.036	Tangible service factor is stronger for international guests.
Cleanliness of rooms and yoga halls	0.047	0.079	0.031	0.056	0.086	0.027	0.007	Marginally stronger for international guests.
Comfortable, well-equipped rooms and ambience	0.062	0.144	0.001	0.057	0.139	0.002	−0.005	Equally important for both groups.
Meals are healthy, fresh, and cater to dietary preferences	0.067	0.161	0	0.074	0.168	0	0.007	Similar significance across both groups.
Staff are courteous, helpful, and attentive	0.094	0.141	0.001	0.068	0.105	0.014	−0.036	Stronger for Indian guests, empathy-based service.
A peaceful and scenic location enhances relaxation	0.046	0.07	0.042	0.065	0.101	0.018	0.031	Slightly stronger among international guests.
Meditation and mindfulness programs	0.097	0.153	0	0.108	0.172	0	0.019	Important for both, slightly stronger internationally.
Customized wellness programs	0.029	0.071	0.072*	0.043	0.092	0.056*	0.021	Moderately valued across groups.
Reservation and check-in process	0.112	0.155	0	0.103	0.151	0.001	−0.004	Equal weightage among both groups.
Cultural and spiritual experiences	0.046	0.071	0.038	0.06	0.093	0.02	0.022	More valued by international guests.
Post-retreat guidance and resources	0.109	0.145	0	0.123	0.161	0	0.016	Slightly higher for international guests.
**Model R** ^**2**^	**0.71**			**0.76**				The model explains a greater amount of variance among international guests.

### 4.6 Recommendations, implications, and future research

Managerial Suggestions

The primary managerial suggestions focus on enhancing core wellness services, which have the greatest impact on guest satisfaction and exhibit strong positive disconfirmation. These include the quality of yoga classes and meditation courses, the food, and the continuity of wellness after the retreat. As these aspects consistently exceed guests’ expectations, regardless of their cultural backgrounds, management must focus on systematic certification of yoga instructors and lifelong professional development. Explicit presentation of educator qualifications and customised instructional strategies may further promote the perceived quality of service and change above-average performance to high service provision.

Meditation programmes can be enhanced by incorporating qualified teachers from diverse traditions, introducing skills-based tracks (for novices and advanced practitioners), and offering specialised meditation classes tailored to guests’ diverse interests. Such diversification can increase competitive advantage, as there are strong satisfaction effects associated with the quality of meditation. In the same manner, the quality of meals must combine Ayurvedic dietary principles with personalised meal plans tailored to individual constitutions, as well as wellness-oriented culinary education to help guests maintain healthy habits even after the retreat.

Online libraries of yoga, recorded guided meditations, and personalised wellness programs should be used to augment post-retreat wellness continuity and promote long-term change after the retreat experience. Secondary recommendations emphasise the need to differentiate along cultural lines. To appeal to Indian guests, who represent the largest share of the market, the management needs to focus on empathy-based service, relationship-building, and individual interaction with guests by consulting with them daily and conducting wellness checks. For international guests, it is more important to focus on professional certifications, open qualifications, standardised services, and digital service improvements to be reliable and trusted.

### 4.7 Theoretical and practical implications

In theory, the results can be generalised to extend the SERVQUAL framework, as they indicate that the dimensions of service quality have different impacts on customer satisfaction, with core wellness services having a more significant impact than peripheral tangible characteristics. The research paper also supports Hofstede’s cultural dimensions theory, as the researchers discover that collectivist Indian guests are more interested in interpersonal warmth and service based on relationships, whereas international guests are more individualistic and focus on professionalism and apparent service quality. In a practical sense, the findings indicate that differentiation in wellness tourism should be based on the quality of core wellness services rather than luxurious facilities, because transformational outcomes are more significant in satisfying customers than peripheral facilities.

### 4.8 Limitations and future research

The study has some limitations, including its cross-sectional and one-destination designs and the use of self-report measures of satisfaction, which could limit generalisability and introduce bias in responses. The cultural classification of Indians and international people also ignores intra-group heterogeneity. The methods that may be considered in future research include longitudinal designs to investigate the dynamics of satisfaction and loyalty over time, the use of individual-level cultural scales, and comparative research across different wellness destinations to deepen understanding of global and context-specific satisfaction processes.

## Ethical approval statement

This study did not involve any sensitive personal data collection, clinical procedures, or interventions. This study adhered to the Declaration of Helsinki and the ICMR National Ethical Guidelines (2017) (ICMR, 2017), Table 4.2, Exemption from Review. Link. https://ethics.ncdirindia.org/asset/pdf/ICMR_National_Ethical_Guidelines.pdf. According to institutional norms and guidelines for social science research, ethical approval was not required for this type of study. No personally identifiable information was collected at any stage, ensuring respondents’ complete anonymity.

## Data Availability

Figshare: Service Quality and Cross-Cultural Guest Satisfaction in Wellness Tourism Evidence from Yoga Retreats in India’s Global Wellness Destination. https://doi.org/10.6084/m9.figshare.31095796.v2. ( Yadav & Nagendra et al., 2026). This project includes the following underlying data:
• Data set.xlsx Figshare: Service Quality and Cross-Cultural Guest Satisfaction in Wellness Tourism Evidence from Yoga Retreats in India’s Global Wellness Destination. https://doi.org/10.6084/m9.figshare.31095796.v2. ( Yadav & Nagendra et al., 2026). This project contains the following extended data:
• Questionnaires_Wellness.pdf Data are available under the terms of the Creative Commons Attribution 4.0 International license (CC-BY 4.0).
