# Supplementary material for: Service quality and cross-cultural guest satisfaction in wellness tourism: evidence from yoga retreats in India’s global wellness destination
Source: F1000Res. 2026 Apr 16;15:541. [Version 1] doi: 10.12688/f1000research.177368.1 (PMC13215356; doi:10.12688/f1000research.177368.1)
Supplement: Peer review history

# Reviewer report: Reviewer response for version 1

**DOI:** 10.5256/f1000research.195582.r476486

**Published:** 2026-04-30

**Authors:** Sidharth Srivastava

**Affiliations:** 1 School of Hospitality & Tourism Galgotias University, Noida, Uttar Pradesh, India

**Introduction and Contributions**

 The paper provides a valuable and timely investigation into the rapidly growing sector of wellness tourism, specifically focusing on Rishikesh, India. By utilizing an integrated SERVQUAL and Expectation-Confirmation Theory (ECT) framework, the authors explore the nuances of service quality and its impact on guest satisfaction across domestic (Indian) and international cohorts. The study's primary contribution lies in addressing the research gap regarding culturally moderated satisfaction formation in a globally recognized yoga hub, offering practical insights for destination managers to tailor their service strategies.

**Strengths of the Work**

- **Methodological Rigor**: The adoption of a quantitative, cross-sectional design with a robust sample size of 289 participants provides a solid empirical foundation. The use of paired sample t-tests and multiple regression analyses allows for a sophisticated understanding of both expectation-perception gaps and the drivers of satisfaction.
- **Thematic Relevance**: The paper effectively links theoretical frameworks like Hofstede’s cultural dimensions with practical service attributes. Identifying that Indian guests prioritize empathy and interpersonal warmth while international guests emphasize tangibles and efficiency is a significant finding that adds depth to the existing literature.
- **Clear Practical Implications**: The authors translate their statistical findings into actionable recommendations for the wellness industry, such as prioritizing staff empathy training for domestic markets and enhancing environmental and digital efficiency for international guests.

**Suggestions for Improvement**

 While the paper presents valuable insights, it would benefit from a few minor adjustments to further enhance its clarity and impact:

- **Clarification on Qualitative Dimensions**: The paper notes that wellness experiences are often "intangible, experiential, and transformative". It might be beneficial to include a brief discussion or a future research suggestion on how qualitative methods could complement these quantitative findings to capture the "spiritual transformation" aspect more deeply.
- **Discussion of Outliers**: In the analysis of service attributes, core services like yoga instruction and meal quality showed non-significant gaps between expectations and perceptions. The paper could benefit from further clarification on why these "universal drivers" remain consistent across cultures compared to the highly variable empathy or tangible dimensions.
- **Visual Representation of Data**: Consider revising the section on demographic distribution by incorporating a summary chart (e.g., a bar graph for age or gender distribution) to allow readers to grasp the participant profile more quickly than via Table 2 alone.
- **Expanding the Literature Review**: While the current review is thorough, the authors might consider including more recent global studies from 2024–2025 to ensure the "rapidly growing" nature of the segment is reflected with the most contemporary data available.

Overall, this is a well-executed and insightful study. By implementing these minor changes, the authors can further strengthen a paper that already makes a substantial contribution to the field of wellness tourism management.

Is the work clearly and accurately presented and does it cite the current literature?

Yes

If applicable, is the statistical analysis and its interpretation appropriate?

Yes

Are all the source data underlying the results available to ensure full reproducibility?

Yes

Is the study design appropriate and is the work technically sound?

Yes

Are the conclusions drawn adequately supported by the results?

Yes

Are sufficient details of methods and analysis provided to allow replication by others?

Yes

Reviewer Expertise:

Tourism and Hospitality

I confirm that I have read this submission and believe that I have an appropriate level of expertise to confirm that it is of an acceptable scientific standard.

# Reviewer report: Reviewer response for version 1

**DOI:** 10.5256/f1000research.195582.r476490

**Published:** 2026-04-24

**Authors:** I Gusti Bagus Rai Utama

**Affiliations:** 1 Universitas Dhyana Pura, Bali, Indonesia

Overall, there is strong consistency among the title, abstract, objectives, theory, methods, results, conclusions, and recommendations, and the manuscript is suitable for acceptance after **minor revision** focused on clarity and tightening, not on design or analysis.

**1. Title, abstract, and objectives**

- The title “Service quality and cross-cultural guest satisfaction in wellness tourism: evidence from yoga retreats in India’s global wellness destination” accurately reflects the core constructs (service quality, cross‑cultural satisfaction) and the empirical context (yoga retreats in Rishikesh).
- The abstract clearly states the research gap (little work comparing Indian vs international guests), the theoretical frame (integrated SERVQUAL and Expectation–Confirmation Theory), the method (quantitative, cross‑sectional survey of 289 guests, t‑tests, multiple and multi‑group regression), main findings (positive disconfirmation, cultural differences in key dimensions), and contributions.
- The four objectives in the Introduction match the abstract and are specific and testable: expectation–perception gap, most important attributes, cross‑cultural differences, and recommendations.

**Suggestion:**

- Very minor: In the abstract, add 1–2 short phrases to state explicitly the three hypotheses (H1–H3) or at least “three hypotheses based on SERVQUAL and ECT were tested” so readers see the inferential focus from the beginning.

**2. Theory, conceptual framing, and hypotheses**

- The literature review sections (1.1–1.6, 2, 3) systematically build from service quality and satisfaction in wellness tourism to ECT and cross‑cultural perspectives, leading logically to the research gaps.
- The use of SERVQUAL (tangibles, reliability, responsiveness, assurance, empathy) and Expectation–Confirmation Theory is appropriate for expectation–perception gaps and satisfaction formation; this is clearly articulated.
- The cross‑cultural framing (Hofstede, value–satisfaction–loyalty models) is consistent with the objective to compare Indian vs international guests and underpins H1–H3 logically.
- The research gaps and hypotheses (H1, H2, H3) are explicitly stated and directly connected to the literature and to the planned analyses (gaps, drivers, cultural moderation).

**Suggestion:**

- The phrase “to examine the absence of the expectation–perception gap” is contradictory to the later finding of positive disconfirmation (there is a gap); re‑phrase as “to examine the expectation–perception gaps, satisfaction determinants…” to keep wording consistent.

**3. Methods and results alignment**

- The methods clearly describe a quantitative, cross‑sectional, comparative design with purposive stratified sampling of five yoga retreats and spa resorts in Rishikesh, and N=289 (178 Indian, 111 international).
- Measurement is consistent with SERVQUAL and ECT: expectations and perceptions on a five‑point Likert scale, five dimensions, and 15 service attributes; this aligns very well with the objectives and hypotheses.
- The analyses described (paired t‑tests for expectation–perception gaps, independent/multi‑group tests and regressions for cross‑cultural differences and satisfaction drivers) are exactly those reported in Tables 2–5 and in the narrative results.
- Results are reported in a way that maps directly to the objectives:
  - Objective 1 (gaps): Table 3 and associated text on positive disconfirmation across 12 of 15 attributes.
  - Objective 2 (satisfaction drivers): overall regression (R²=0.705) and coefficients in Table 4.
  - Objective 3 & H3 (cross‑cultural differences): multi‑group regression (R²=0.71 vs 0.76) and Table 5.

**Suggestions (minor):**

- Clarify briefly how “expectation–perception gaps” were operationalized (e.g., P–E difference scores) and confirm that higher positive scores mean positive disconfirmation, to help readers interpret Table 3 easily.
- The text around Table 3 is very long and interpretive; consider breaking it into shorter paragraphs and moving some detailed interpretations (e.g., exact mean differences for each attribute) into a succinct summary paragraph or into an appendix to improve readability.

**4. Results, conclusions, and recommendations**

- The conclusions (4.6–4.8 and closing paragraphs) are firmly grounded in the reported results: they emphasize that core wellness services (yoga, meditation, spa, food) are the strongest satisfaction drivers and that empathy vs tangibles differ by culture, exactly as shown in Tables 3–5.
- Managerial suggestions (e.g., emphasis on empathy and relationship‑building for Indian guests, and professional expertise, environmental quality, digital efficiency, and cultural immersion for international guests) follow directly from the multi‑group regression findings and the t‑test patterns.
- Theoretical implications (extension of SERVQUAL and ECT, support for Hofstede’s cultural dimensions) are consistent with the empirical results and with the stated theoretical framework.
- Limitations (cross‑sectional design, single destination, self‑report, broad “Indian vs international” categorization) are appropriate and match the methods, and the proposed future research (longitudinal, other destinations, individual‑level cultural measures) is reasonable.

**Suggestions (minor wording and structure):**

- In 4.7 and 4.8, separate clearly:
  - theoretical implications (for SERVQUAL, ECT, cultural theories),
  - practical implications (how managers should design services),
  - and limitations/future research, possibly using short sub‑headings or bullet points, to make these sections easier to scan.
- Some very long sentences in the recommendations and implications sections could be shortened to enhance clarity, especially for non‑native readers.

**5. Overall coherence and small issues**

- Coherence: There is high internal consistency among title, abstract, objectives, theory, methodology, results, conclusions, and recommendations; each section supports the others, with no major contradictions.
- The statistical reporting (R, R², adjusted R², F, p‑values, beta coefficients) is coherent across text and tables, and interpretations (e.g., 70.5% variance explained, stronger effects for some attributes) are appropriate for the numbers shown.
- Ethics and data availability statements are compatible with the described design (non‑sensitive, anonymous survey; no formal ethics review required; dataset and questionnaire provided in Figshare).

**Minor technical/language points you might correct:**

- Clean up a few duplicated or awkward phrases such as “The original Theory of Expectation‑Confirmation Theory and Satisfaction Formation” and “absence of the expectation‑perception gap” for stylistic precision.
- Check all in‑text citations for consistent formatting (e.g., “Leou & Wang., 2023” → “Leou & Wang, 2023”) and ensure every reference appears in the list.

**6. Final decision**

 Given:

- Strong alignment between title, abstract, objectives, theory, methods, results, conclusions, and recommendations.
- Clear, coherent hypotheses and analytical strategy that directly address the research gap.
- Robust and well‑reported statistical results with appropriate interpretation and practical implications.
- Only minor issues related to wording, redundancy, and clarity, not to scientific design or analysis.

My editorial recommendation is: **Accept with minor revision** (language/clarity and small consistency edits, not further methodological or analytical changes).

Is the work clearly and accurately presented and does it cite the current literature?

Yes

If applicable, is the statistical analysis and its interpretation appropriate?

Yes

Are all the source data underlying the results available to ensure full reproducibility?

Yes

Is the study design appropriate and is the work technically sound?

Yes

Are the conclusions drawn adequately supported by the results?

Yes

Are sufficient details of methods and analysis provided to allow replication by others?

Yes

Reviewer Expertise:

Tourism Management

I confirm that I have read this submission and believe that I have an appropriate level of expertise to confirm that it is of an acceptable scientific standard.
